# Promoting HIV, Hepatitis B Virus, and Hepatitis C Virus Screening Among Migrants With a Language Barrier: Protocol for the Development and Evaluation of an Electronic App (Apidé)

**DOI:** 10.2196/22239

**Published:** 2021-05-05

**Authors:** Frédérique Thonon, Saleh Fahmi, Olivia Rousset-Torrente, Pascal Bessonneau, James W Griffith, Carter Brown, Olivier Chassany, Martin Duracinsky

**Affiliations:** 1 Patient-Reported Outcomes Unit (PROQOL), UMR 1123, Université de Paris, INSERM, F-75004 Paris France; 2 Unité de Recherche Clinique en Economie de la Santé (URC-ECO), AP-HP, Hôpital Hôtel-Dieu, F-75004 Paris France; 3 Department of Medical Social Sciences, Feinberg School of Medicine Northwestern University Chicago, IL United States; 4 Service de Médecine Interne et d'Immunologie Clinique Hôpital Bicêtre, F94270 Le Kremlin-Bicêtre France

**Keywords:** HIV, hepatitis B, hepatitis C, migrants, screening, language barrier, eHealth

## Abstract

**Background:**

Late diagnoses of HIV, hepatitis B, and hepatitis C are important public health problems that affect the population at large and migrants in particular. Missed opportunities of HIV and hepatitis screening are numerous, with language differences being a significant barrier to testing. Several studies have shown that migrants who do not speak the language of the health provider are less likely to get tested, due to health providers’ reluctance to offer a test and to migrants’ reluctance to accept testing.

**Objective:**

The aim of our study is to develop a multilingual electronic tool (app) that assists health providers in offering and explaining HIV and hepatitis screenings to migrants with a language barrier and to evaluate its acceptability and impact in terms of public health.

**Methods:**

The study will go through 3 stages: (1) concept development, (2) app development, and (3) app evaluation. A qualitative study has been undertaken to explore language barriers during health care encounters and their effect on communication, specifically when a screening test is offered. In parallel, a systematic review of the literature was conducted to have a comprehensive overlook of electronic tools designed to help health care providers communicate with migrants with a language barrier. To generate a list of items to be translated for inclusion in the app, we will conduct a focus group and Delphi survey. The development of the app will include translation and voice recording of items. The electronic development will also include 3 steps of user testing. The acceptability of the app will be evaluated using the System Usability Scale. Evaluation of the app’s efficacy will consist of a stepped wedge randomized controlled trial. The study will be carried out in 16 centers that treat migrants and offer them screening tests for infectious diseases. The primary outcome is the percentage of screening tests realized. The secondary outcomes are the rate of screening proposal by health professionals, acceptance rate by migrants, number of positive cases using this app, and frequency of use of the app.

**Results:**

The app evaluation study received a 3-year grant from the Agence Nationale de la Recherche contre le SIDA et les hépatites virales (ANRS) and from the Office Français de l’Immigration et Intégration (OFII). At the time of publication of this protocol, the initial qualitative study and systematic literature review were completed.

**Conclusions:**

This study will develop an app that assists health providers in offering and explaining HIV and hepatitis screenings to migrants with a language barrier and measure its acceptability and effectiveness in terms of public health. When completed, this app could be distributed to numerous professionals carrying out screening with migrant populations in various health care settings.

**International Registered Report Identifier (IRRID):**

PRR1-10.2196/22239

## Introduction

There are currently around 150,000 persons living with HIV in France, and late diagnosis is an important public health problem. In addition to the individual therapeutic benefit, early diagnosis contributes, according to modeling, to primary infection prevention [1,2]. Early antiretroviral treatment initiation inhibits the multiplication of the virus, reducing the viral load and therefore the risks of future contamination. This strategy, however, requires extended screening [3,4]. Migrants (people born outside France and of non-French nationality at birth) are a population particularly at risk of the late diagnosis of HIV, as well as hepatitis B and C [5-7]. Migrants are also more prone to late diagnosis than nonmigrants [8-10]. The disproportionate risk of contracting HIV infection that migrants face in their host country is likely the result of a combination of factors, including stigma, increased risky behavior, and limited access to HIV prevention services [8]. Among the reasons for late diagnosis, the language barrier for non-French–speaking migrants might play an important role.

Research has been extensively conducted to investigate the language barrier among migrants in accessing care and prevention services and their consequences on health. Specifically, qualitative and quantitative studies carried out among different population of migrants in Australia [11], Canada [12,13], and England [14] found that lack of proficiency in English is a barrier to accessing testing for either HIV or hepatitis B or C. Those results have been confirmed in studies of health professionals caring for migrant patients in Belgium [15], England [14], and Australia [16] who state language barrier as a reason for not offering a screening test for those infectious diseases.

Beyond language proficiency issues, migrants may also face health literacy issues. The World Health Organization defines health literacy as “the cognitive and social skills which determine the motivation and ability of individuals to gain access to, understand and use information in ways which promote and maintain good health” [17]. Health literacy can aid a person in making informed choices, reducing health risks, and improving their quality of life [18]. Thus, health literacy is important to understand to what extent the communication barrier is due to the patient's low level of health literacy in their mother tongue and to what extent it is due to the language difference.

The STRADA study (Screening strategies for infectious diseases [tuberculosis, HIV, hepatitis C, hepatitis B] in the migrant population in France) is a current, ongoing, prospective, multicenter, observational study to assess the effectiveness of a strategy for screening HIV, hepatitis B virus (HBV), and hepatitis C virus (HCV) among migrants undergoing a medical examination at the French Office for Immigration and Integration (OFII) [19]. Eligible migrants undergoing a medical examination at OFII are offered rapid screening for the 3 viruses. During the informed consent process, participants are informed that the study is voluntary and independent from the residency permit. This screening is preceded by a short risk factor questionnaire, available in 11 languages (French, English, Arabic, Mandarin Chinese, Bengali, Russian, Lingala, Portuguese, Spanish, Turkish, Haitian Creole). This screening is not mandatory, and individuals are given the option to decline or accept such testing. Migrants who are invited to participate receive information in their own language; this information indicates that they are free to refuse this test, that their refusal to participate will not change anything regarding their residency permit or their level of care (during the medical check-up or in the future), that the participation and results of the tests are kept confidential and separate from administrative papers, and that in case of a positive result, they will be able to access free treatment in France. It is made clear to the participant that a positive result is not grounds for refusing a residency permit. Throughout STRADA, we set up a study of acceptability aimed at measuring the obstacles impeding screening so as to identify strategies for achieving a better acceptance rate. A 3-minute online form is filled out by the health practitioners to report practitioners’ reasons for not offering and patients’ motives for refusing testing [20-22]. The results from over a year show the proposition and acceptability rates are 87.1% and 49.9%, respectively, of the patients who attended the medical check-up. Impeded communication is reported in 29.6% of the reasons health professionals are not offering screening tests, of which 93.6% are related to language barriers (see Table 1). In addition, other cited obstacles to communication include illiteracy, the presence of informal translators (which might hinder screening where sensitive and confidential information is shared), or a general lack of understanding. Among migrants who are offered screening, a majority who refuse (38.0%) said they have already been screened, although health professionals do not mention if they asked when the last screening was performed or checked thoroughly if the screening was indeed performed for the 3 diseases (HIV, HBV, HCV). Communication barriers and low health literacy might make it difficult for health professionals to investigate whether migrants need a repeat screening. Impeded communication is reported in 7.5% of the reasons migrants refuse screening when it is offered. Other reasons for refusing screening include patients do not want screening or do not see any relevance (19.2%), and 5.2% feel either not at risk or not concerned (Table 2). Those reasons might be linked to a lack of knowledge regarding HIV and hepatitis risks and necessity for regular testing, which might be overcome with clear explanations by a health professional.

Overall, communication is a major issue in the implementation of screening, and, as such, a linguistic app targeted at improving how screening is proposed to patients could potentially meet an identified need, by reducing the language barrier on one hand and by providing motivational content to health care workers to help them explain the benefits of testing on the other hand.

**Table 1:** Reasons health professionals are not offering screening.

Reason stated	Responses, %
Organizational issues	36.6
Language, communication	29.6
Not known	10.8
Pregnancy	6.1
Already screened	3.9
Lack of time	3.9
Technical problems	3.6
Religious	3.2
Other	2.4

**Table 2:** Reasons migrants refuse screening when it is offered.

Reason stated	Responses, %
Already screened	38.0
Not interested	19.2
Not known	14.9
Language, communication	7.5
No reason given	5.8
No risk factor or not concerned	5.2
Lack of time	4.0
Other reasons	4.0
Religious	1.3

Health care professionals use multiple solutions to communicate with migrants with a language barrier: informal interpreters, formal interpreters in person or over the phone, and general translation applications. Informal interpreters are a no-cost solution that enables building a relationship of trust but represent a burden for patients who have to adjust to their availability. Informal interpreters also commit more errors with serious clinical consequences [23,24] and do not allow frank communication [25,26]. Professional interpreters provide high-quality translations that result in a better quality of care [23,27]. Medical doctors and patients are generally satisfied with professional interpreters but are limited by their availability and number of languages [26]. Professional interpreters over the phone offer the same quality of translation with greater time flexibility and more languages. However, health care professionals report less satisfaction [28,29], longer consultation times [28], and more technical problems [29] than with in-person interpreters. Although less expensive than in-person interpreters, this service still has a cost that cannot be met by various health organizations [30].

General translation applications (eg, Google Translate, Lexilogos, iTranslate) have the advantage of being free of charge and available 24/7. Some studies have tested their efficacy in the medical context with mixed results. Studies have found the quality of translation ranging from similar to professional translation [31] to incomprehensible [32] or potentially dangerous [33], depending on the specific application and language tested. The translation of languages that are widely spoken tended to be of better quality than less widespread languages. Online translation apps are not appropriate for long or complex sentences [31], for critical situations, [34], in case of emergency, or if consent is needed [35]. They are also time consuming [36], not appropriate to use with patients who cannot read, and do not allow patients to respond [30].

Besides the general translation applications mentioned earlier, some applications or electronic tools have been specifically developed to be used in medical consultations to facilitate the dialogue between health care professionals and migrants with low language proficiency. Those medical translation applications have developed different features to overcome such drawbacks. They usually consist of a list of sentences commonly used in a medical setting, translated into several languages, either by bilingual researchers or by professional translators. Those sentences are often supported by an audio recording and/or culturally adapted pictures to illustrate them. Patients can rarely give feedback on the health professional’s sentences. Given the quantity and diversity of sentences that can be used in a medical setting, those applications are usually targeted to a specific medical setting, such as patient’s assessment [37]; emergency medicine [38] or anesthesia [39]; specific pathologies, such as asthma [40]; or a specific population, such as refugees [41-43]. No existing application has been developed to facilitate communication between migrants with a language barrier and health professionals regarding testing for HIV, HBV, and HCV.

The aim of the Apidé study (Electronic Application to promote screening among Migrants) is to develop and evaluate an app to assist with screening for HIV, HBV, and HCV among migrants who have low French proficiency. This will include a databank of phrases to offer, explain, and conduct a test for HIV, HBV, and HCV screening in several languages, with a voice version and pictograms, sociocultural adaptation, and adaptation to the patient's level of literacy. It will include a short evaluation questionnaire and items adapted according to the level of understanding. It will help health professionals test migrants who do not speak French and/or have a low level of health literacy. The tool will also include motivational content to improve acceptability, depending on the situation. For example, a frequent reason for migrants' refusal to screen was a lack of risk perception. The app will help overcome this objective using motivational content and explanations of the risks associated with a failure to be screened.

## Methods

**Figure 1:**
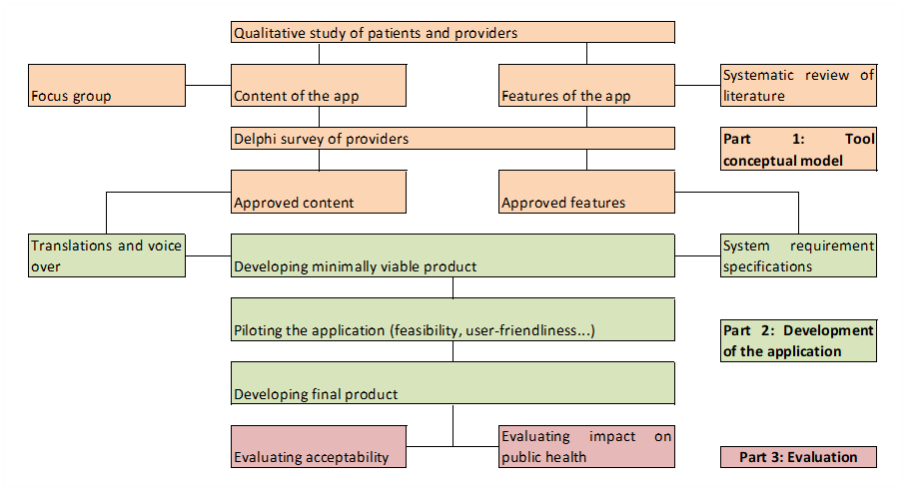
Overview of the steps in the Apidé project.

The study will take place in 3 parts that are detailed in the following paragraphs: development of the conceptual model, development of the app, and evaluation of the app (Figure 1). The approach we use is similar to the agile methodology for developing software, which is centered on users’ satisfaction and collaboration and consists of 10 stages: communication (with potential users), requirements gathering (from the demands of users), feasibility study, system analysis (of limitations and impact), software design and coding, testing , integration (of the different modules of the software), implementation (on users’ computers), operation and maintenance, and disposition [44]. In our study, the users are primarily health care workers (doctors, nurses, midwives) and other professionals involved in migrants’ testing, as they will use the app for themselves and guide migrants to use it.

In the first part of the project, we will develop the tool’s conceptual model based on a qualitative study of migrants and health care providers, a systematic review of the literature on electronic apps for migrants with a language barrier, and a focus group and modified Delphi survey with health care providers who propose screening tests to migrants. After conducting those different steps, we will have the app conceptual model, as well as language selection and a proposed layout for the app.

In the second part of the project, we will develop the app. The conceptual model will be translated by a service provider with experience in translation with cultural adaptation. All sentences will be voice-recorded in the chosen languages. The app will be developed, followed by a pilot study of the acceptability and usability of the app.

In the third part of the project, we will evaluate the acceptability and efficacy of the tool. The acceptability will be evaluated using the System Usability Scale (SUS) [45]. The efficacy of the tool will be evaluated in an epidemiological study set up in various settings where migrants go for medical consultations: OFII medical check-up, free hospital consultations (Permanences d’accès aux soins de santé [PASS]), and charity outreach that offers rapid tests. The chosen methodology is a stepped wedge randomized trial [46,47].

### Qualitative Study

A qualitative study was undertaken to explore language barriers during health care encounters and their effect on communication with health care professionals, specifically in the situation when a screening test (HIV or hepatitis) is offered. The language barriers were analyzed in relation to the cultural background of the interviewed migrants and their past screening experiences in their country of origin. Participants were all legal migrants who were present at OFII in order to undergo the obligatory routine medical visit needed to validate their residency permit. The study has been completed, and the results have been analyzed [48,49] for publication in a peer-reviewed journal. This study provides feedback from the expected user cohort on the content and features of the application.

### Systematic Review of the Literature

Developing the app requires an in-depth knowledge of existing electronic tools facilitating communication between migrants and health professionals, the methods used to develop them, and available data on their acceptability and efficacy, including evidence on whether specific features or options improve the efficacy or acceptability. The objective of our systematic review of the literature is to have a comprehensive overview of electronic tools designed to help health care providers communicate with migrants who have low proficiency in the language of the country of destination or a low level of health literacy and critically synthesize evidence about the acceptability and efficacy of those electronic tools. We have written a protocol prior to starting the search, established inclusion and exclusion criteria, and evaluated the quality of selected articles. The database search, study selection, and data extraction were independently performed by 2 researchers, and the results were reported according to the PRISMA (Preferred Reporting Items for Systematic Reviews and Meta-Analyses) standard for reporting systematic reviews. This systematic review will help create an overview of existing apps helping to improve health communication with migrants, as well as identify the best features of the app for improving screening.

### Focus Group and Delphi Survey

In order to generate a list of items and phrases to be translated and added to the electronic tool, we will use the following steps. First, we will create a list of suggested sentences used to offer, explain, and carry out a screening test. It will be informed by current guidelines on testing [50-54]. This list will be enriched by findings from the qualitative study. Then, we will conduct focus groups with health professionals. Focus groups are group interviews that include a moderator and an observer, where questions and items are being discussed in an interactive way. We will organize 2-3 focus groups of 20 individuals who have experience or expertise in testing migrants for HIV, HBV, and HCV. Unlike the qualitative study, which discussed general themes about communication with migrants with a language barrier, the focus groups will specifically discuss the text suggested by the literature to be used in the app. Health care professionals will provide suggestions for adaptation to a low level of literacy and adaptation to different cultures. Once the focus groups have been completed, their results will be used to enrich the initial list. We will also create a separate list of technical features of the app that could be incorporated. This list will be created from findings of the systematic review and will be enriched by findings from the qualitative study. The final list of items to be included in the electronic tool will be selected with a modified Delphi survey. The modified Delphi technique is a structured process that uses a series of questionnaires or “rounds” to gather information. Rounds are held until group consensus is reached [55,56]. The panel of invited participants will include a large panel of health care professionals treating migrants or conducting HIV, HBV, and HCV testing, as well as migrants’ community organizations. At the end of this process, we will have a databank of phrases to be included the app.

### Preparation of the App

Following those steps, we will have a validated conceptual model in the form of a pilot in French, with the content and features of the app approved (ie, a minimally viable product).

The pilot's items will be translated by a service provider with experience in translation with cultural adaptation. The choice of the languages to include in the app will be determined in the Delphi survey previously described.

Voice recordings in languages will be produced. Then, a pilot version of the electronic tool will be developed. Development of the app will be guided by system requirement specifications (SRS) that will be written by our team.

### Piloting of the App

This first version will be tested in 3 stages: First, the research team will test the first version according to its intended use. The team will test all the languages and audio versions and all different combinations of sentences and different scenarios of use, as well as the technical quality of the app. All feedback, problems, and difficulties will be carefully documented and provided to the app developers.

Second, the revised app will be tested among health professionals outside real-life situations. We will invite a small but diverse sample (5-10 health professionals representing all intended categories of users) to try the app in the presence of the research team. Each session will be audio-recorded; users will be invited to navigate the app and comment aloud on its ease of use, ergonomics, and user-friendliness. All comments will be transcribed, then compiled and sent back to the developer’s team for adjustments. This piloting will be performed with the Cognitive Walkthrough approach, which is a method used to evaluate the design of a user interface [57]. It is often used for the development of health care information systems and is recommended for infrequent or inexperienced users [58].

Then, the third version will be tested in real-life situation by health professionals and migrants. A large sample of health professionals will be provided with an electronic tablet or set up to use their own computer, depending on the situation. All participants will receive a demonstration of the app’s functioning. Health providers will be invited to use the app during consultations with migrants and then will give specific feedback in the form of cognitive debriefing.

During the second and third steps, we will use cognitive interviews and cognitive debriefing. Cognitive interviewing is traditionally a key technique in uncovering potential problems with survey questionnaires through a process of administering draft survey questions and then probing how subjects comprehend, recall, decide, and respond to the questions [59]. Although this technique is primarily for questionnaire development, it is also relevant for software usability testing [60]. Cognitive debriefing consists of the use of both verbal probing by the interviewer and think aloud in which the interviewer asks the respondent to verbalize whatever comes to mind as he or she answers the question [61].

### Evaluation of the Acceptability

The acceptability of the electronic tool will be evaluated in a survey with the SUS questionnaire in French. The SUS is a simple, 10-item scale giving a global view of subjective assessment of usability. It was constructed from a pool of 50 potential questionnaires [45]. The original SUS instrument is composed of 10 statements that are scored on a 5-point scale of strength of agreement. Final scores for the SUS range from 0 to 100, where higher scores indicate better usability.

The advantages of the SUS compared with other similar instruments are that it is flexible enough to assess a wide range of interface technologies; the survey is relatively quick and easy to use by both study participants and administrators; the survey provides a single score on a scale that is easily understood by the wide range of people (from project managers to computer programmers) who are typically involved in the development of products and services; and the survey is nonproprietary, making it a cost-effective tool [62]. Given the diversity of potential users of the app, a large sample of potential users is necessary to evaluate its acceptability. Nielsen and Molich [63] recommend a maximum of 30 users; therefore, we aim to have a sample of 30 participants for this study. The participants will be the future users of the app; therefore, the sample of participants will consist of health professionals (doctors, nurse, midwives), volunteers, and other health care workers involved in the screening of migrants.

### Evaluation of the Efficacy

#### Justification of Analysis

The study design for evaluating the tool will be a stepped wedge, randomized controlled trial. In this design, an intervention is launched sequentially to clusters over a number of time periods. This design is characterized by the fact that clusters are randomized for the time at which the cluster will switch from the control condition to the intervention condition. Stepped wedge designs incorporate data collection at each point (step) where a new group receives the intervention [46]. The period with no intervention is used as the control, which allows the study to take into account independent variables that might affect screening rate (such as migration changes or behavior changes brought on by the COVID-19 pandemic). This pragmatic design enables evaluations of how interventions would work in a real-world setting with limited exclusion criteria [64]. The other reason for choosing a stepped wedge cluster is ethics; it could be unethical or politically controversial to withhold an intervention that is deemed more effective than harmful to a control cluster [47]. The stepped wedge methodology is often used in public health research projects, especially in the field of HIV [47].

#### Setting

The study will be carried out in 16 centers taking care of and treating migrants, including the provision of screening tests: immigration centers (OFII), hospital consultations specialized for underserved persons (PASS), nonprofit organizations offering rapid tests to migrants (not exclusively), ambulatory health care professionals (general practitioners, gynecologists, midwives) with large migrant patient populations. Different regions of France will be represented.

#### Inclusion and Exclusion Criteria

For participating centers, the inclusion criteria are as follows. For the types of settings, OFII centers having participated in the STRADA study, PASS hospital services, medical doctors or midwives, and organizations accredited to conduct HIV and hepatitis B and C rapid tests will be used. Regarding the level of experience of the centers, we will require that they be consulting with a significant number of migrants (≥500 the previous year), experienced in offering those screening tests to migrants, and experienced in orienting patients with a positive result to an appropriate hospital service and in orienting patients to other support services if needed. Centers must also have the following resources and organization: have sufficient resources to get a phone interpreter or solution in case of a positive result and sufficient privacy to conduct a test with the assistance of an audio app.

For individual participants (migrants), the inclusion criteria are migrant status (born outside of France), over 18 years of age, having a low level of proficiency in French (not able to follow the consultation alone), not speaking another language in common with the health professional, and fluently speaking a language available in the app. The exclusion criteria are being French, speaking French, or not able to understand any of the languages available in the tool.

#### Intervention and Control

The control period will correspond to the typical processes of these centers for screening migrants (use of phone or in-person interpreters, informal interpreters, or not offering screening tests to migrants because of the language barrier). The intervention will be the use of the app during consultations with non-French speaking migrants to offer, explain, and carry out the screening test, with the possible help from professional interpreters if necessary.

#### Outcomes

The primary outcome measured is the percentage of screening tests administered. The secondary outcomes are rate of screening proposals by health professionals or associate workers, acceptance rate by migrants, number of positive cases during screening using this app, and frequency of use of the app.

#### Sample Size

The overall rate of screening currently carried out as part of the STRADA study in OFII centers is around 45% (average of 2 years, taking into account health professionals not offering a test and migrants refusing when the screening is offered). The primary hypothesis of this study is that the addition of the electronic screening app will increase this rate by 10%, from 45% to 55% (including an increase in the rate of screening offered by health professionals and a decrease in the refusal rate among migrants to whom screening is offered). With an alpha risk of 0.05 and a beta risk of 0.20, the total number of subjects required is 778 (ie, a workforce of 900 participants to be included to take account for missing data). Statistical comparisons will be made at the threshold of *P*<.05.

A moderate improvement was chosen because it would be a realistic but meaningful improvement, whereas a small effect size would be too small for clinical significance. Thus, our sample size will allow for the detection of moderate improvements, with ample data for subgroup analyses as well.

We will do a simple and pragmatic step wedge with 2 groups and 3 steps: first step without intervention, second starting intervention for 1 group (half of the centers), and third all centers with the intervention [65,66]. We plan to have 2 clusters with 450 participants in the OFII centers and 450 participants in the other structures (PASS, associated structures). Power and sensibility fit sample size and step wedge configuration were checked with the design effect method [67,68].

Subgroup analyses will be performed based on several variables (screening structure, level of health literacy, geographic origin of migrant, level of education, gender, age group) without adjusting the significance threshold.

#### Enrollment and Data Collection

At the beginning of the study, all participating investigators will have a briefing session about the aims of the study and the enrollment process and instructions. During the control period, the participants will receive a quick daily questionnaire to calculate the number of eligible patients seen in consultation and the number of patients to whom a screening test is offered and accepted (see Multimedia Appendix 1).

#### Statistical Analysis

We will use a mixed effects approach that allows computing effects with step wedge [69]. Mixed models will allow us to evaluate effects of treatment and covariates in one model.

#### Reporting the Results

The results will be published according to the validated reporting guidelines for stepped wedge randomized trials, which is the CONSORT (Consolidated Standards of Reporting Trials) guideline with the specialized extension [70]. A specialized CONSORT extension for the reporting of eHealth clinical trials has been created [71], which we will also use. We will present the 2 reporting guidelines as separate attachments.

#### Ethical Aspects and Possible Risks

The study will be submitted to the appropriate authorities: Comité de Protection des Personnes d’Ile de France (institutional review board) and Commission Nationale de l’Informatique et des Libertés (data protection agency). And the protocol will be registered in a clinical trials database [72].

As the study will be conducted among providers who are already experienced in conducting HIV and hepatitis B and C tests, the risks related to the testing and referral are minimal.

One potential risk is the introduction of a test to a population of patients who would not have been tested because of language barriers: patients not understanding the results of a test and having either a false sense of security or unjustified panic, receiving a positive result being distressed and the provider not able to comfort them because of language barriers, or receiving a positive result and not fully understanding the need for follow-up appointment.

To prevent and control these potential risks, we will only include centers that have sufficient resources to call for a phone interpretation service in case of a positive result or difficult communication.

Other risks specific to the use of an app to aid the communication around testing relate to confidentiality and data security. For example, there could be a lack of confidentiality if the audio version is played aloud and other people can hear from an adjacent room. It should be noted that this risk already exists for other consultations where health care professionals and migrants have a language barrier and use a phone interpreter service on a loudspeaker. There could also be a lack of data confidentiality if the sentences related to the results of the tests are recorded during the app session.

To prevent and control this risk, investigators conducting the study will be instructed to only use the app on a fixed computer or tablet securely kept (locked in a secure building) with a user password. During the development of the app, we will ensure that data security concerns are emphasized in the SRS. Sessions will be erased after a short period of time.

## Results

The study has received preliminary financing from the Agence Nationale de la Recherche contre le SIDA et les hépatites virales (French Agency for Research against AIDS and viral Hepatitis [ANRS]), followed by 1 year of funding from the Office Français de l’Immigration et Intégration (French agency for migration and integration [OFII]) and then by 3-year funding, including doctoral funding from ANRS. Further funding has been requested from private sponsors and Région Ile-de-France.

At the time of publication of this protocol, the initial qualitative study and systematic literature review have been completed. The study has been completed, and the results have been analyzed [48,49,73] for publication in a peer-reviewed journal. For the qualitative study, we interviewed 33 migrants undergoing a medical check-up at OFII. Migrants reported that difficult communication with a French doctor resulted in lack of confidence and lower compliance with treatment. Migrants with a French-speaking partner were either sidelined during the medical visits, being completely dependent on translation, or their partners helped them in learning new words needed for the medical visits. For migrants who preferred translation, the preference between physically present interpreters versus interpretation by phone or the use of applications was influenced by mainly 4 factors: perceived quality of translation (interviewees were divided, with some perceiving a human interpreter with knowledge of medical jargon most accurate and others trusting the application more), trust, intimacy, and empathy.

For the systematic review, we collected general information about the app, information about health literacy and cultural adaptation, information about the development of the app, evidence about the app’s acceptability and efficacy, and information about the use of apps. We included 61 articles presenting a total of 48 applications. About one-third of the applications (16/48) were designed solely to facilitate the interaction between migrants and a health care provider during a consultation, while the remaining two-thirds (32/48) were designed to promote health among migrants with a language barrier. Overall, the applications had good levels of acceptability, while only half had their efficacy evaluated. In those evaluations, the endpoints used are mostly related to reported behavior change and knowledge improvement, which is common for evaluations of health promotion programs.

Focus groups and Delphi consensus panel for selection of the tool’s content are currently underway.

## Discussion

### Implications of the Study

The project is well underway, with the qualitative study and systematic reviews completed. Their results will be used for the development of the app and the design of our study. The qualitative study has highlighted the necessity to have professional interpreters announce a positive result, preferably who can be reached easily. The qualitative study has also given us clues on how migrants use translation applications that can be useful for the development of our app: For example, the study found a strong desire to learn new medical terms; this possibility will be considered for the development of the app. Thanks to the systematic review, we have critically reviewed existing translation applications and noted the lessons learnt from their development and recommendations. We have compiled specific lists of features of such applications that are associated with an increased acceptability or efficacy. Those features will be suggested as options to include in our app in our Delphi survey.

If the study demonstrates an increase in screening rates and acceptability by migrants, further development may include international deployment, so as to make this app available for other countries and in other languages. Other developments might include expanding the health promotion features of the electronic tool, such as educational videos and a text messaging follow-up service for migrants who did not wish to undertake the screening immediately but were interested in a screening consultation in the future.

### Strengths and Limitations

The strength of this study is that it is conducted by a research team with multidisciplinary skills, including experience in developing patient-reported outcomes (including electronic patient-reported outcomes). The team has extensive experience with working with migrants within the STRADA study [74] and has worked with many professionals working in migrants’ health or HIV testing, all of whom are included in the steering committee, scientific committee, or associated committee. This study includes several methods (qualitative, quantitative, systematic literature review) and includes users in the development process.

The COVID-19 pandemic has disturbed the advancement of the study in many ways. The research team, specialized in public health and infectious diseases, has been developing research projects related to the pandemic, with a priority that has taken precedence over other existing research projects, including this study. This focus on COVID-19, as well as the postponement of recruiting new staff due to social distancing and remote working requirements and postponement of the focus groups for the same reasons, has delayed the advancement of the study. However, those short delays do not jeopardize the integrity and ultimately, the completion, of our study. In fact, better screening of infectious diseases is all the more necessary in the era of a pandemic.

### Conclusion

This study will develop an electronic screening app to aid migrants who speak little or no French and measure its acceptability and effectiveness in terms of public health. At the end of this project, this app will be able to be distributed to numerous health care workers, including nonprofessionals (such as volunteers), conducting screening with migrant audiences to be used in current practice. If proven effective, the electronic tool will make testing for HIV and hepatitis B and C among migrants more readily available and more widespread and will be an asset in the fight against infectious diseases.

## Appendix

Multimedia Appendix 1 Questionnaire for the control clusters.

Multimedia Appendix 2 Project peer-review report 1.

Multimedia Appendix 3 Project peer-review report 2.

Multimedia Appendix 4 Translation of the peer-review reports.

## Abbreviations

ANRS: Agence Nationale de la Recherche contre le SIDA et les hépatites virales (French Agency for Research against AIDS and viral Hepatitis)
CONSORT: Consolidated Standards of Reporting Trials
HBV: hepatitis B virus
HCV: hepatitis C virus
OFII: Office Français de l’Immigration et Intégration
PASS: Permanences d’accès aux soins de santé
PRISMA: Preferred Reporting Items for Systematic Reviews and Meta-Analyses
SRS: system requirement specifications
STRADA: Stratégie de dépistage de maladies infectieuses (Tuberculose, VIH, VHC, VHB) dans la population des migrants en France (Screening strategies for infectious diseases (Tuberculosis, HIV, Hepatitis C, Hepatitis B) in migrant population in France)
SUS: System Usability Scale
