# Supplementary material for: Promoting HIV, Hepatitis B Virus, and Hepatitis C Virus Screening Among Migrants With a Language Barrier: Protocol for the Development and Evaluation of an Electronic App (Apidé)
Source: JMIR Res Protoc. 2021 May 5;10(5):e22239. doi: 10.2196/22239 (PMC8135028; doi:10.2196/22239)
Supplement: Multimedia Appendix 1 [file resprot_v10i5e22239_app1.docx]

**Appendix 1 : Questionnaire for the control clusters**

| Date | __/__/____ | *Automatic date* |
| --- | --- | --- |
| Center | __________ | *Drop-down menu* |
| Having many patients have been consulting with you today? | *___* | *Number* |
| How many are eligible for a HIV/HBV/HCV screening test? | *___* | *Number* |
| How many patients have been offered a HIV/HBV/HCV screening test? | *___* | *Number* |
| Reasons you didn’t offer a HIV/HBV/HCV screening test | □ Linked to the service organization (lack of time, personnel…) *___* | *Number concerned* |
|  | □ Language or communication barrier *(specify what language) ___* | *Number concerned* |
|  | □ Recently tested (last 3 months) *___* | *Number concerned* |
|  | □ Other reason *___* |  |
| How many patients have accepted HIV/HBV/HCV screening test? | *___* | *Number* |
| Reasons the migrants refused HIV/HBV/HCV screening test | □ Already tested *___* | *Number concerned* |
|  | □ Not interested *___* | *Number concerned* |
|  | □ Language or communication barrier *___* | *Number concerned* |
|  | □ Not feeling at risk *___* | *Number concerned* |
|  | □ Doesn’t have the time *___* | *Number concerned* |
|  | □ Others *___* | *Number concerned* |
| How many patients in total had a language barrier | *___* | *Number* |
| Have you used any solution to communicate with them? | None *___* | *Number concerned* |
|  | Phone interpreter*___* | *Number concerned* |
|  | In-person interpreter *___* | *Number concerned* |
|  | Informal interpreter (family, friend) *___* | *Number concerned* |
|  | Translation application *___* | *Number concerned* |
