# Supplementary material for: Promoting HIV, Hepatitis B Virus, and Hepatitis C Virus Screening Among Migrants With a Language Barrier: Protocol for the Development and Evaluation of an Electronic App (Apidé)
Source: JMIR Res Protoc. 2021 May 5;10(5):e22239. doi: 10.2196/22239 (PMC8135028; doi:10.2196/22239)
Supplement: Multimedia Appendix 2 [file resprot_v10i5e22239_app2.pdf]

ANRS - GRILLE D'EVALUATION Projet - AO

Demandeur	Expert
M. CHASSANY Olivier	Rapporteur A

Titre : Application électronique d'aide au dépistage chez les personnes migrantes (ApiDé)

PARTIE I : Evaluation Scientifique

- **Le projet relève-t-il des missions scientifiques de l'ANRS ?** **oui**

- **Qualité du projet (scientifique et technique)**

Pertinence pour la recherche sur l'infection à VIH, les hépatites virales et/ou co-infections	**Excellent**
Progrès par rapport à l'état actuel des connaissances	**Bon**
Définition des hypothèses et des objectifs	**Bon**
Pertinence de l'approche méthodologique, statistique et/ou technologique	**Excellent**
Impact potentiel du projet	**Bon**

- **Faisabilité du projet**

La durée du projet est-elle raisonnable pour sa réalisation ?	**Bon**
Environnement scientifique et ressources du laboratoire (collaboration, missions/déplcaements, conditions de sécurité)	**Bon**

PARTIE II : Adéquation budget / projet

- **Coûts**

Fonctionnement	**Raisonnable**
Equipement (< 16 000 euros HT)	**Raisonnable**
Personnel	**Raisonnable**
Participation demandée à l'ANRS	**Raisonnable**
Coût total estimé du projet	**Raisonnable**

PARTIE III : Dimension éthique

Les problèmes éthiques ont-ils été pris en considération (homme, animal) ?	**Oui**
Si le projet relève de la règlementation applicable à la recherche sur la personne, les demandes nécessaires ont-elles été prises en considération?	**Oui**

PARTIE IV : Respect de la charte d'éthique de la recherche dans les pays en développement

	**Ce sujet est-il abordé dans le projet ?**	**Si oui, de façon satisfaisante ?**
L'impact potentiel de la recherche pour la collectivité en terme de santé publique est-il envisagé ?	**Oui**	**Excellent**
Le rapport bénéfice-risque pour la personne participante est-il évalué ?	**Oui**	**Bon**
Des moyens pour assurer la confidentialité sont-ils pris ?<br>(confidentialité liée à la séropositivité, aux données personnelles, …)	**Oui**	**Bon**
Un médecin référent sera-t-il désigné pour chaque participant ?	**Oui**	**Bon**
La constitution d'un comité indépendant est-elle prévue ?	**NonApplicable**	**NonApplicable**
Des moyens pour éviter les conséquences discriminatoires ou stigmatisantes de la recherche sont-ils pris ?	**Oui**	**Bon**
Un counselling pré et post test de dépistage est-il prévu ?	**Oui**	**Bon**
La prise en charge médicale pendant la recherche est-elle assurée ?<br>(par le projet, par le système de santé du pays, etc…)	**Oui**	**Bon**
Les conditions de prise en charge post-recherche sont-elles définies ?	**Oui**	**Bon**
Des moyens pour communiquer les résultats de la recherche aux participants sont-ils définis ?	**Oui**	**Bon**
Les bénéfices de la recherche seront-ils rendus accessibles à la personne participante ?	**Oui**	**Bon**
Des représentants qualifiés de la communauté ou des associations de personnes vivant avec le VIH ou une hépatite virale sont-ils impliqués dans la mise en place et le déroulement de ce projet de recherche ?	**Oui**	**Moyen**
Si une notice d'information et/ou un formulaire de consentement sont fournis, leurs contenus vous paraissent-ils adaptés ?	**Non**	**Insuffisant**

RAPPORT

Le dépistage reste un des points faible des stratégies de contrôle de l'infection VIH surtout chez les migrants dont une grande proportion ne connait pas leur statut vis-à-vis du VIH. Cette population vulnérables à de grandes difficultés d'accès soins d'abord physiques du fait de contraintes financières ou administrative mais également du fait de contraintes sociales comme les difficultés de communication avec le personnel de santé.

Ce travail fait suite à l'étude STRADA qui a évalué efficacité de stratégie de dépistage chez les migrants et montré la contribution de la barrière de la langue aux difficultés d'accès aux soins dans cette population. L'objectif de ce travail est donc de développer une application multilinguistique et multiculturelle pour aider les soignants à proposer et expliquer le dépistage du VIH et des hépatites auprès des d'un public allophone et d'évaluer l'acceptabilité et l'impact de cet outil.

Points forts

- Cette question porte sur la population vulnérable des migrants qui est l'une des principales contributrices à la persistance du VIH du fait de la prévalence VIH élevée et du faible taux de dépistage dans cette population. C'est une population cibles prioritaires pour l'ANRS
- Le projet est très bien écrit avec un argumentaire convainquant se basant sur des résultats très intéressants de l'étude STRADA financée par l'ANRS. Il est innovant par l'utilisation de nouvelles technologies pour améliorer le dépistage dans une population difficile d'accès. A la différence des outils de traduction existants qui n'intègre pas suffisamment de termes, l'approche de développement proposée voudrait pallier à cette limite en en associant pleinement les soignants à toutes les étapes.
- En méthodes, les étapes proposées sont assez cohérentes. (i) Une première étape de développement du concept de l'outil en se basant sur les résultats de l'étude STRADA chez des migrants, une revue de la littérature, des focus groupe et une enquête auprès des soignants, (ii) deuxième étape de développement de l'outil incluant une phase de test et acceptabilité ; (iii) et une dernière étape d'évaluation de l'impact de l'outil dans le cadre d'essai randomisé en Stepped wedge.
- L'équipe du projet à une grande expérience en recherche sur le VIH et les Hépatites comme en témoigne le travail en cours avec l'étude STRADA.

Points faibles

- L'outil sera utilisé pour aider à la communication entre les soignants et les migrants mais la phase de développement implique très peu les migrants. En effet la première étape du concept se basera sur les résultats d'étude qualitative chez les migrants mais la deuxième phase ne semble pas impliquer les migrants. La sélection des phrases à traduire devrait plus faire intervenir les migrants ou association de migrants qui pourraient aider à rendre l'outil plus interactif. En effet, une conception ne prenant en compte que le point de vue des soignants conduira au développement d'un outil plutôt d'information que de communication.
- Cet outil aura selon les auteurs l'avantage d'intégrer des termes médicaux comparativement à des outils plus génériques comme Google translate. Mais contrairement aux l'outil génériques qui dispose de ressources importantes pour la maintenance et les mises à jour, comment l'outil sera-t-il mis à jour en termes de nouvelles langues, nouvelles phrase… ? Cet aspect même si certainement liée à la mesure de l'impact, l'efficacité de l'outil, devrait être un peu plus développé.

CONCLUSION

Ce projet porte sur question de recherche prioritaire et propose d'utilisation d'un outil innovant. De plus, il sera mené par une équipe expérimentée dont les travaux précédemment financés ont produit des résultats très intéressants à l'origine de ce nouveau projet. C'est un projet très bien écris et cohérent dont les résultats pourront grandement aider au dépistage VIH chez les migrants.

AAvisPrioritaire
