# Supplementary material for: Promoting HIV, Hepatitis B Virus, and Hepatitis C Virus Screening Among Migrants With a Language Barrier: Protocol for the Development and Evaluation of an Electronic App (Apidé)
Source: JMIR Res Protoc. 2021 May 5;10(5):e22239. doi: 10.2196/22239 (PMC8135028; doi:10.2196/22239)
Supplement: Multimedia Appendix 3 [file resprot_v10i5e22239_app3.pdf]

ANRS - GRILLE D'EVALUATION Projet - AO

Demandeur	Expert
M. CHASSANY Olivier	Rapporteur B

Titre : Application électronique d'aide au dépistage chez les personnes migrantes (ApiDé)

PARTIE I : Evaluation Scientifique

✶ **Le projet relève-t-il des missions scientifiques de l'ANRS ?** **oui**

✶ **Qualité du projet (scientifique et technique)**

Pertinence pour la recherche sur l'infection à VIH, les hépatites virales et/ou co-infections	**Bon**
Progrès par rapport à l'état actuel des connaissances	**Bon**
Définition des hypothèses et des objectifs	**Moyen**
Pertinence de l'approche méthodologique, statistique et/ou technologique	**Bon**
Impact potentiel du projet	**Moyen**

✶ **Faisabilité du projet**

La durée du projet est-elle raisonnable pour sa réalisation ?	**Bon**
Environnement scientifique et ressources du laboratoire (collaboration, missions/déplcaements, conditions de sécurité)	**Bon**

PARTIE II : Adéquation budget / projet

✶ **Coûts**

Fonctionnement	**Raisonnable**
Equipement (< 16 000 euros HT)	**Raisonnable**
Personnel	**Raisonnable**
Participation demandée à l'ANRS	**Raisonnable**
Coût total estimé du projet	**Raisonnable**

PARTIE III : Dimension éthique

Les problèmes éthiques ont-ils été pris en considération (homme, animal) ?	**Oui**
Si le projet relève de la règlementation applicable à la recherche sur la personne, les demandes nécessaires ont-elles été prises en considération?	**Non**

PARTIE IV : Respect de la charte d’éthique de la recherche dans les pays en développement

	**Ce sujet est-il abordé dans le projet ?**	**Si oui, de façon satisfaisante ?**
L’impact potentiel de la recherche pour la collectivité en terme de santé publique est-il envisagé ?	**Oui**	**Insuffisant**
Le rapport bénéfice-risque pour la personne participante est-il évalué ?	**Oui**	**Bon**
Des moyens pour assurer la confidentialité sont-ils pris ?<br>(confidentialité liée à la séropositivité, aux données personnelles, …)	**Oui**	**Bon**
Un médecin référent sera-t-il désigné pour chaque participant ?	**Oui**	**Bon**
La constitution d’un comité indépendant est-elle prévue ?	**Oui**	**Bon**
Des moyens pour éviter les conséquences discriminatoires ou stigmatisantes de la recherche sont-ils pris ?	**Oui**	**Bon**
Un counselling pré et post test de dépistage est-il prévu ?	**Non**	**NeSaitPas**
La prise en charge médicale pendant la recherche est-elle assurée ?<br>(par le projet, par le système de santé du pays, etc…)	**Oui**	**Bon**
Les conditions de prise en charge post-recherche sont-elles définies ?	**Oui**	**Bon**
Des moyens pour communiquer les résultats de la recherche aux participants sont-ils définis ?	**Oui**	**Bon**
Les bénéfices de la recherche seront-ils rendus accessibles à la personne participante ?	**Oui**	**Bon**
Des représentants qualifiés de la communauté ou des associations de personnes vivant avec le VIH ou une hépatite virale sont-ils impliqués dans la mise en place et le déroulement de ce projet de recherche ?	**Oui**	**Bon**
Si une notice d’information et/ou un formulaire de consentement sont fournis, leurs contenus vous paraissent-ils adaptés ?	**Non**	**Insuffisant**

Rapport:
Ce projet d'une durée de 36 mois, qui est accompagné d'une demande de financement de thèse, fait suite à un contrat d'initiation porté par l'équipe « Patient-Reported-Outcomes de l'INSERM à l'hôtel-Dieu, pour lequel un avis favorable avait été émis par la CSS 14 lors du 2ème appel d'offre de 2019.
Le projet vise à développer et évaluer l'acceptabilité d'une application multilingue et multiculturelle pour aider au dépistage du VIH et des hépatites chez les migrants allophones, et enfin d'évaluer son potentiel impact pour la santé publique.
Pas de cofinancement acquis, les financements initialement demandés auprès de l'ARS PACA est également prévu pour financer le projet et tester l'outil auprès des acteurs de la région PACA et auprés de la DGOS fin juillet 2019 pour financer l'intégralité du projet, n'ont pas été obtenus. Des financements ont été demandé auprès de la région IDF et de Gilead (Getting to zero).
Le porteur du contrat d'initiation est également le porteur de l'étude STRADA, dont l'objectif est d'évaluer l'efficacité d'une stratégie de dépistage du VIH et des hépatites B et C chez les migrants effectuant la visite médicale à l'Office Français de l'Immigration et de l'Intégration (OFII). Les migrants éligibles qui effectuent la visite médicale à l'OFII se voient proposer un dépistage par test rapide (TROD) des trois virus, indépendamment du parcours administratif. Ce dépistage est précédé d'un court questionnaire de facteurs de risque (TROD screen).
Le porteur du projet part du constat que les barrières linguistiques peuvent être un frein à la proposition et à l'acceptation de tests de dépistage. Dans l'étude STRADA, les barrières linguistiques représentent une raison fréquente de non proposition et de refus de dépistage. Quelques études dans la littérature ont également souligné le fait que les barrières linguistiques pouvaient être un frein à l'accès aux soins et à la prise en charge.
Le porteur évoque également le niveau de littératie en santé chez les migrants, et souligne l'importance de comprendre dans quelle mesure la barrière de communication est due au faible niveau de littératie en santé chez la personne (i.e. le niveau de compétences des personnes pour comprendre, évaluer et utiliser les informations et les concepts de santé afin de faire des choix éclairés, de réduire les risques pour la santé et d'améliorer leur qualité de vie) et dans quelle mesure elle est due à la différence de langue.
A ce jour, plusieurs solutions sont utilisées par les médecins pour les consultations avec les patients ne parlant pas français, telles que des outils papier et guide spécifique, des applications de traduction (Google translate), les interprètes informels (famille, amis, etc), les interprètes professionnels physique, l'interprétariat professionnels par téléphone ; le recours à des interprètes professionnels a un coût qui peut être difficilement supportable pour les petites structures. A cela s'ajoute un outil électronique complémentaire traducmed.fr, qui propose un ensemble d'enregistrements de phrases et de questions fermées spécifiques à une consultation médicale, mais rien pour le dépistage du VIH et des hépatites.
Le porteur propose alors de développer et tester un outil informatique afin d'augmenter le taux de dépistage du VIH, VHB et VHC chez les migrants, allophone qui ne parle aucune langage commune avec leur interlocuteurs de santé.
Pour cela, il y aura une revue de la littérature visant à répertorier les outils de traduction et d'aide à la consultation médicale. Puis une étude qualitative, sous forme d'entretiens semi-directif, avec un volet auprès des migrants, pour décrire les difficultés rencontrés par les migrants lors des consultations médicales, décrire ses préférences pour l'offre de dépistage et l'intérêt pour la présence d'un interprète, et évaluer le niveau de littératie en santé, et un second volet auprès d'intervenants du dépistage, afin d'explorer les attentes des soignants en ce qui concerne un outil de dépistage et de recueillir des phrases clés qui sont utilisées lors de consultation de dépistage.
Cette étude qualitative sera suivi d'un sondage Delphi afin de sélectionner les phrases utilisées lors de la consultation de dépistage. Le contrat d'initiation a permis de réaliser l'étude quantitative et la revue de la littérature et il reste à faire le focus group et une étude Delphi. Ces trois étapes permettront de développer le concept d'un outil informatique, et est en fait la première étape du

projet.
Ensuite un modèle conceptuel de l'application en français sera validé, puis des traductions et des enregistrements vocaux seront réalisés dans plusieurs langues ; pour certaines langues des pictogrammes accompagneront certaines phrases, et une adaptation culturelle sera réalisée. L'application sera potentiellement ajustée après avoir été testé par des professionnels de santé et auprès de migrants.
Enfin l'acceptabilité de cette application et l'impact de cette application sur le taux de dépistage sera évalué via un essai randomisé en grappes avec permutation séquentielle (stepped wedge cluster randomised trial) impliquant 16 centres qui reçoivent des migrants. Ce sont les migrants qui ne parlent pas un langage commun avec le professionnel de santé (OFII, PASS, associations) qui seront invités à participer à l'étude. Le critère de jugement principal sera le pourcentage de dépistage réalisés, et les critères secondaires seront le pourcentage de proposition et le pourcentage d'acceptation et le nombre de positifs trouvés lors du dépistage. Il faudra inclure 900 sujets, sous l'hypothèse que l'application permettra d'augmenter le taux de dépistage de 10%, soit de 45% à 55%.
L'application sera téléchargeable sur mobile offline. L'application comportera une boite de dialogue qui proposera des réponses en fonction des réponses et des préférences de l'interlocuteur. Ces réponses aideront à proposer, expliquer et à amener l'interlocuteur à accepter un test pour le VIH, VHC et VHB, en plusieurs langues, en version orale et avec des pictogrammes, une adaptation socio-culturelle et une adaptation prenant en compte le niveau de littératie, donc l'application comprendra un test de littératie.
Commentaires :
Des outils/moyens permettant d'aider au dépistage du VIH et des hépatites chez les migrants allophones pourraient permettre à chacun et à chacune de se voir proposer un test de dépistage du VIH et des hépatites au moins une fois dans la vie comme le recommande actuellement la Haute Autorité de Santé, et potentiellement de réduire les opportunités manquées de diagnostics dans ces populations.
Toutefois, l'impact sur la réduction des opportunités manquées de diagnostics, sur la découverte de séropositivité, et donc l'impact en Santé Publique, dépendra surtout du risque d'infection par le VIH et les hépatites de la partie des migrants qui sont allophones. Les porteurs du projet prévoit d'évaluer cet impact en santé publique, mais aucun détail sur la méthodologie n'est donné. Comparé à la soumission du contrat d'initiation, les porteurs du projet ont rajouté le pourcentage de test de dépistage qui n'ont pas été proposé par les soignants à cause de la barrière linguistique (30%), et le pays de naissance des personnes qui n'ont pas eu de test proposé à cause de la barrière linguistique. Or l'évaluation de cet impact sera dépendant du pourcentage de positifs parmi les personnes allophones et du pourcentage d'allophone parmi les migrants, ou autrement dit du pourcentage d'allophones parmi les personnes diagnostiquées avec le VIH ou une hépatite en France. Mais ces éléments sont absents de la description du projet, donc il est difficile d'apprécier le potentiel impact en Santé Publique de cette nouvelle application.
Comme indiqué précédemment, l'étude de la littératie en santé chez les migrants (i.e. l'étude du niveau de compétences des personnes pour comprendre, évaluer et utiliser les informations et les concepts de santé afin de faire des choix éclairés, de réduire les risques pour la santé et d'améliorer leur qualité de vie) est un élément intéressant et original de ce projet, qui mériterait d'être approfondie. Le porteur laisse entendre que les résultats de l'étude de la littératie sera pris en compte dans le développement de l'application pour améliorer la communication entre le soignant et la personne migrante lors de la proposition de dépistage. Par contre, il n'est prévu de questions de recherche ou bien d'interventions pour accroitre le niveau de compétences des personnes pour comprendre, évaluer et utiliser les informations et les concepts de santé afin par exemple d'accroitre leur accès aux services de santé après la visité médicale ou le test de dépistage sera proposé.
Il est indiqué que l'intérêt de l'outil c'est d'expliquer les risques liés au VIH et hépatites en fonction du niveau de littératie. Il est à plusieurs reprises indiqué dans la description du projet qu'un des freins à la proposition du dépistage chez les soignants est le rendu du résultats positifs. En ce qui concerne les personnes migrantes, des craintes étaient liées au fait d'avoir les résultats du test immédiatement et

à la fiabilité perçues du test, la question de la confiance. Est-ce que cette application va prendre en compte ces freins qui limitent la proposition et l’acceptation du dépistage ?

Au final, ne faut-il pas généraliser cette application à l’ensemble des migrants, y compris ceux qui parlent le français ou une langue commune avec l'interlocuteur de santé, et y inclure des informations épidémiologiques sur la prévalence du VIH et des hépatites dans chaque pays et zone géographie, ainsi que des délais entre infection et diagnostic, et expliquer en quoi une absence de diagnostic est une perte de chance pour l’individu, etc. Cela permettrait d’objectiver ces risques et conséquences pour les personnes migrantes et pour les interlocuteurs de santé d’utiliser l’application comme support pour proposer le test.

CONCLUSION

Avis favorable avec quelques réserves sur le possible impact de l’application en Santé publique et sur l’absence de question de recherche annexe à l’évaluation d’un application numérique pour améliorer le taux d’acceptation des tests de dépistage.

NOTATION GENERALE

BAvisFavorable
