# Supplementary material for: Promoting HIV, Hepatitis B Virus, and Hepatitis C Virus Screening Among Migrants With a Language Barrier: Protocol for the Development and Evaluation of an Electronic App (Apidé)
Source: JMIR Res Protoc. 2021 May 5;10(5):e22239. doi: 10.2196/22239 (PMC8135028; doi:10.2196/22239)
Supplement: Multimedia Appendix 4 [file resprot_v10i5e22239_app4.docx]

# Multimedia Appendix 4 : Translation of the peer-review reports

### Rapporteur A

| **Part 1 : Scientific evaluation** |  |
| --- | --- |
| *** Does the project fall under the scientific missions of the ANRS?** | **Yes** |
| *** Quality of the project (scientific and technical)** |  |
| Relevance for research on HIV infection, viral hepatitis and / or co-infections | Excellent |
| Progress compared with the current state of knowledge | Good |
| Definition of hypothesizes and objectives | Good |
| Relevance of the methodological, statistical and / or technological approach | Excellent |
| Potential impact of the project | Good |
| Project’s feasibility |  |
| Is the duration of the project reasonable for its completion? | Good |
| Scientific environment and laboratory resources (collaboration, missions / travel, security conditions) | Good |

| **Part 2 : Budget / project adequacy** |  |
| --- | --- |
| *** Costs** |  |
| Operation | Reasonable |
| Equipment | Reasonable |
| Staff | Reasonable |
| Requested participation from ANRS | Reasonable |
| Total estimated cost for the project | Reasonable |

| **Part 3 : Ethical aspects** |  |
| --- | --- |
| Have ethical issues been taken into consideration (humans, animals)? | Yes |
| If the project falls under the regulations applicable to research on humans, have the legal considerations been taken into consideration? | Yes |

| **Part 4: Respect for the research ethics charter in developing countries** | **Is this subject**  **addressed in the**  **project?** | **If so, is it addressed in a satisfactory manner?** |
| --- | --- | --- |
| Is the potential impact of research for the community in terms of public health considered? | Yes | Excellent |
| Is the benefit-risk ratio for the participant assessed? | Yes | Good |
| Are means taken to ensure confidentiality? (confidentiality linked to HIV status, personal data, etc.) | Yes | Good |
| Will a referring doctor be appointed for each participant? | Yes | Good |
| Is the establishment of an independent committee planned? | Not applicable | Not applicable |
| Have means to avoid the discriminatory or stigmatizing consequences of research been taken? | Yes | Good |
| Is there pre- and post-test counseling available? | Yes | Good |
| Is medical care during the research ensured? (by the project, by the country's health system, etc.) | Yes | Good |
| Are the post-research support conditions defined? | Yes | Good |
| Are there ways of communicating research results to participants defined? | Yes | Good |
| Will the benefits of the research be made accessible to the participant? | Yes | Good |
| Are qualified representatives of the community or associations of people living with HIV or viral hepatitis involved in the setting up and progress of this research project? | Yes | Average |
| If an information notice and/or consent form is provided, is the content suitable? | No | Insufficient |

**Report**

Screening remains one of the weak points of HIV infection control strategies, especially among migrants, a large proportion of whom do not know their HIV status. This population is vulnerable to major difficulties in accessing physical health care due to financial constraints: administrative, but also because of social constraints such as difficulties in communication with health personnel.

This work follows the STRADA study which assessed the effectiveness of a screening strategy in migrants and showed the contribution of the language barrier to difficulties in accessing healthcare in this population. The objective of this work is therefore to develop a multilingual and multicultural application to help health professionals offer and explain HIV and hepatitis testing with an allophone public and to assess the acceptability and impact of this tool.

Strong points:

- This question concerns the vulnerable migrant population which is one of the
  major contributors to the persistence of HIV due to the high HIV prevalence and
  the low screening rate in this population. It is a priority target population
  for the ANRS.
- The project is very well written with a convincing rationale based on
  very interesting results from the STRADA study funded by the ANRS. It is innovative by
  the use of new technologies to improve screening in a population
  difficult to access. Unlike existing translation tools which do not integrate
  enough terms, the proposed development approach would like to overcome this
  limit by fully involving health professionals at all stages.
- In terms of methods, the proposed steps are fairly consistent. (i) A first step in
  development of the concept of the tool based on the results of the STRADA study
  among migrants, a review of the literature, focus groups and a survey of
  health professionals, (ii) second stage of tool development including a test phase and
  acceptability; (iii) and a final step of evaluating the impact of the tool with a
  randomized stepped wedge trial.
- The project team has extensive experience in HIV and Hepatitis research
  as evidenced by ongoing work with the STRADA study.

Weaknesses:

- The tool will be used to help communication between health professionals and migrants but
  the development phase does not involve migrants much. Indeed the first step of
  concept will be based on the results of qualitative study among migrants but the second
  phase does not seem to involve migrants. The selection of sentences to translate should
  more involving migrants or migrants' association that could help to make
  the more interactive tool. Indeed, a design taking into account only the point of view
  of caregivers will lead to the development of an information tool rather than
  communication.
- According to the authors, this tool will have the advantage of integrating medical terms compared to more generic tools like Google translate. But unlike the generic tool which has significant resources for maintenance and updates, how will the tool be updated in terms of new languages, new sentences…? This aspect although certainly linked to the measure of the impact, the effectiveness of the tool, should be developed a little more.

**Conclusion**

This project addresses a priority research question and proposes the use of an innovative tool. In addition, it will be led by an experienced team whose previously funded work produced very interesting results, which led to this new project. It is a very well written and coherent project, the results of which will greatly assist in screening HIV among migrants.

### Rapporteur B

| **Part 1 : Scientific evaluation** |  |
| --- | --- |
| *** Does the project fall under the scientific missions of the ANRS?** | **Yes** |
| *** Quality of the project (scientific and technical)** |  |
| Relevance for research on HIV infection, viral hepatitis and / or co-infections | Good |
| Progress compared with the current state of knowledge | Good |
| Definition of hypothesizes and objectives | Average |
| Relevance of the methodological, statistical and / or technological approach | Good |
| Potential impact of the project | Average |
| Project’s feasibility |  |
| Is the duration of the project reasonable for its completion? | Good |
| Scientific environment and laboratory resources (collaboration, missions / travel, security conditions) | Good |

| **Part 2 : Budget / project adequacy** |  |
| --- | --- |
| *** Costs** |  |
| Operation | Reasonable |
| Equipment | Reasonable |
| Staff | Reasonable |
| Requested participation from ANRS | Reasonable |
| Total estimated cost for the project | Reasonable |

| **Part 3 : Ethical aspects** |  |
| --- | --- |
| Have ethical issues been taken into consideration (humans, animals)? | Yes |
| If the project falls under the regulations applicable to research on humans, have the legal considerations been taken into consideration? | No |

| **Part 4: Respect for the research ethics charter in developing countries** | **Is this subject**  **addressed in the**  **project?** | **If so, is it addressed in a satisfactory manner?** |
| --- | --- | --- |
| Is the potential impact of research for the community in terms of public health considered? | Yes | Insufficient |
| Is the benefit-risk ratio for the participant assessed? | Yes | Good |
| Are means taken to ensure confidentiality? (confidentiality linked to HIV status, personal data, etc.) | Yes | Good |
| Will a referring doctor be appointed for each participant? | Yes | Good |
| Is the establishment of an independent committee planned? | Yes | Good |
| Have means to avoid the discriminatory or stigmatizing consequences of research been taken? | Yes | Good |
| Is there pre- and post-test counseling available? | No | Don’t know |
| Is medical care during the research ensured? (by the project, by the country's health system, etc.) | Yes | Good |
| Are the post-research support conditions defined? | Yes | Good |
| Are there ways of communicating research results to participants defined? | Yes | Good |
| Will the benefits of the research be made accessible to the participant? | Yes | Good |
| Are qualified representatives of the community or associations of people living with HIV or viral hepatitis involved in the setting up and progress of this research project? | Yes | Good |
| If an information notice and/or consent form is provided, is the content suitable? | No | Insufficient |

**Report**

This 36-month project, which is accompanied by a request for PhD funding, is following an initiation contract carried by the "Patient-Reported-Outcomes team of INSERM at the Hôtel-Dieu, for which a favorable opinion was issued by CSS 14 during the 2nd call for proposals of 2019. The project aims to develop and assess the acceptability of a multilingual and multicultural application to help screening for HIV and hepatitis in allophone migrants, and finally to assess their potential impact on public health.

No co-funding acquired, the funding initially requested from Regional Health Agency of PACA is also planned to finance the project and test the tool with stakeholders in the PACA region and from the Ministry of Health at the end of July 2019 to finance the entire project, were not obtained. Funding has been requested from the Parisian region and private sponsors.

The project coordinator is also the coordinator of the STRADA study, the objective of which is to assess the effectiveness of a screening strategy for HIV and hepatitis B and C among migrants undergoing the medical examination at the French Office for Immigration and Integration (OFII). Eligible migrants who undergo the medical visit at OFII are offered a rapid test screening for the three viruses, separate from the administrative procedure. This screening is preceded by a short questionnaire of risk factors.

The project leader starts from the observation that language difference can be a barrier to offering and accepting screening tests. In the STRADA study, language barrier represent a frequent reason for not offering and refusing screening. Some studies in the literature have also highlighted the fact that language barriers can be an obstacle to access to care and support.

The coordinator also mentions the level of health literacy among migrants, and underlines the importance of understanding to what extent the communication barrier is due to the weak level of health literacy in the person (i.e. the level of skills of people for understand, assess and use health information and concepts to make choices informed, reduce health risks and improve their quality of life) and to what extent it is due to the language difference.

To date, several solutions are used by doctors for consultations with patients not speaking French, such as paper tools and specific guide, translation applications (Google translate), informal interpreters (family, friends, etc.), professional interpreters physical, professional interpreting by telephone; the use of professional interpreters has a cost which may be difficult to bear for small structures. To add to those tools is an electronic website traducmed.fr, which offers a set of recordings of sentences and closed questions specific to a medical consultation, but nothing for HIV testing and hepatitis.

The coordinator then proposes to develop and test a computer tool in order to increase the rate of
HIV, HBV and HCV screening among migrants, allophone who does not speak any common language
with their health professional.

To that end, the project team will carry out a review of the literature aiming to list the translation and assistance tools used for medical consultation. Then a qualitative study, in the form of semi-structured interviews, with a section with migrants, to describe the difficulties encountered by migrants during medical consultations, describe their preferences for the screening offer and for the presence of an interpreter, and assess their level of health literacy, and a second component with professionals of screening, in order to explore the expectations of health professionals regarding a screening and collect key phrases that are used during screening consultation.

This qualitative study will be followed by a Delphi survey in order to select the sentences used during
the screening consultation. The initiation contract made it possible to carry out the quantitative study and review of the literature and there remains to be a focus group and a Delphi study. These three steps will develop the concept of a computer tool, and is in fact the first step in the project.

Then a conceptual model of the application in French will be validated, then translations and voice recordings will be made in several languages; for some languages pictograms will accompany certain sentences, and a cultural adaptation will be carried out. The application will potentially be adjusted after being tested by healthcare professionals and with migrants.

Finally, the acceptability of this application and the impact of this application on the screening rate will be evaluated via a randomized trial in clusters with sequential permutation (stepped wedge cluster randomized trial) involving 16 centers which receive migrants. The migrants who do not
speak a common language with the health professional (OFII, PASS, associations) will be invited to participate in the study. The primary endpoint will be the percentage of screening achieved, and the secondary criteria will be the percentage of proposal and the percentage acceptance test and the number of positives found during screening. 900 subjects should be included, under the hypothesis that the application increases the screening rate by 10%, from 45% to 55%. The application will be downloadable on mobile offline. It will include a dialog box who will propose answers based on the answers and preferences of the interlocutor. These answers will help to offer, explain and get the interviewer to accept an HIV test, HCV and HBV, in several languages, in oral version and with pictograms, a socio-cultural adaptation and an adaptation taking into account the level of literacy, so the application will include a literacy test.

Comments:

Tools / resources to help screen allophones migrants for HIV and hepatitis could allow everyone to be offered a screening test for HIV and hepatitis at least once in a lifetime as currently recommended by the High Health Authorities, and potentially reduce missed diagnostic opportunities in these
populations.

However, the impact on reducing missed diagnostic opportunities, on finding HIV positivity, and therefore the impact on public health, will mainly depend on the risk of HIV infection and hepatitis in the part of migrants who are allophones. The project leaders plan to evaluate this impact on public health, but no details on the methodology are given. Compared to submission of the initiation contract, the project leaders added the percentage of testing screening that was not offered by caregivers because of the language barrier (30%), and country of birth of people who have not had a test offered due to language barrier. However, the evaluation of this impact will depend on the percentage of positives among allophone people and the percentage of allophones among migrants, or in other words the percentage of allophones among people diagnosed with HIV or hepatitis in France. But these elements are missing from the project description, so it is difficult to assess the potential impact in Public Health of this new application.

As previously indicated, the study of health literacy among migrants (i.e. the study of skill level of people to understand, assess and use information and health concepts in order to make informed choices, reduce health risks and improve their quality of life) is an interesting and original element of this project, which deserves to be thorough. The coordinator suggests that the results of the literacy study will be taken into account in the development of the application to improve communication between the caregiver and the migrant when the screening proposal is made. However, there are no questions of research or interventions to increase the skill level of people to understand, evaluate and use health information and concepts in order, for example, to increase their access to health services after the medical visit or the screening test will be offered.

It is indicated that the interest of the tool is to explain the risks associated with HIV and hepatitis according to the literacy level. It is repeatedly stated in the project description that one of the barriers to screening among health professionals is the rendering of positive results. Concerning
migrants, fears were linked to having the test results immediately and to the perceived reliability of the test, and trust. Will this application take in account these barriers that limit the proposal and acceptance of screening?

In the end, should we not generalize this application to all migrants, including those who speak French or a common language with the health contact, and include epidemiological information on the prevalence of HIV and hepatitis in each country and geographical area, as well as the delays between infection and diagnosis, and explain why a lack of diagnosis is a loss of opportunity for the individual, etc. This would make it possible to objectify these risks and consequences for migrants and health partners of using the application as a support to offer the test.

**Conclusion**

Favorable opinion with some reservations on the possible impact of the application in public health and on the absence of research question annexed to the evaluation of a digital application to improve the acceptance rate of screening.
